# Mammalian meiotic silencing exhibits sexually dimorphic features

**DOI:** 10.1007/s00412-015-0568-z

**Published:** 2015-12-28

**Authors:** J. M. Cloutier, S. K. Mahadevaiah, E. ElInati, A. Tóth, James Turner

**Affiliations:** The Francis Crick Institute, Mill Hill Laboratory, The Ridgeway, Mill Hill, London, NW7 1AA UK; Institute of Physiological Chemistry, Technische Universität Dresden, Dresden, 01307 Germany

**Keywords:** Meiosis, Meiotic silencing, Oocytes, Epigenetics, Checkpoints, Sex differences

## Abstract

During mammalian meiotic prophase I, surveillance mechanisms exist to ensure that germ cells with defective synapsis or recombination are eliminated, thereby preventing the generation of aneuploid gametes and embryos. Meiosis in females is more error-prone than in males, and this is in part because the prophase I surveillance mechanisms are less efficient in females. A mechanistic understanding of this sexual dimorphism is currently lacking. In both sexes, asynapsed chromosomes are transcriptionally inactivated by ATR-dependent phosphorylation of histone H2AFX. This process, termed meiotic silencing, has been proposed to perform an important prophase I surveillance role. While the transcriptional effects of meiotic silencing at individual genes are well described in the male germ line, analogous studies in the female germ line have not been performed. Here we apply single- and multigene RNA fluorescence in situ hybridization (RNA FISH) to oocytes from chromosomally abnormal mouse models to uncover potential sex differences in the silencing response. Notably, we find that meiotic silencing in females is less efficient than in males. Within individual oocytes, genes located on the same asynapsed chromosome are silenced to differing extents, thereby generating mosaicism in gene expression profiles across oocyte populations. Analysis of sex-reversed XY female mice reveals that the sexual dimorphism in silencing is determined by gonadal sex rather than sex chromosome constitution. We propose that sex differences in meiotic silencing impact on the sexually dimorphic prophase I response to asynapsis.

## Introduction

Meiosis is a dual cell division that halves the chromosome content of diploid germ cells. Defects in meiosis can result in gametes carrying the wrong chromosome number, and therefore the key chromosomal events that precede the meiotic divisions are monitored by surveillance pathways or checkpoints (Burgoyne et al. 2009; Handel and Schimenti 2010; Nagaoka et al. 2012). In mammals, these function at two stages. The first, the prophase I checkpoint, monitors homologous synapsis and recombination, while the second, the spindle assembly checkpoint (SAC), functions later at the metaphase/anaphase I transition and monitors bipolar attachment to the meiotic spindle (Burgoyne et al. 2009; Handel and Schimenti 2010; Nagaoka et al. 2012).

In mammals, most cases of human aneuploidy arise from maternal meiotic errors (Hunt and Hassold 2002; Morelli and Cohen 2005; Nagaoka et al. 2012). A number of distinct aetiological factors contribute to this sex bias. For example, in females, univalent chromosomes can readily form bipolar attachments at the first meiotic division, and in doing so, satisfy the requirements of the SAC (Kouznetsova et al. 2007). It is clear that the SAC is also weaker in females than in males. In males (XY), a univalent X chromosome triggers a robust SAC response, resulting in arrest at metaphase I (Burgoyne et al. 1992). However, in females with X chromosome monosomy (XO females), oocytes can progress through the meiotic divisions despite the presence of a misaligned univalent X chromosome (LeMaire-Adkins et al. 1997). The increased efficiency of the SAC in males is at least in part due to a potentiating effect of the Y chromosome gene *Zfy2* (Vernet et al. 2011).

In contrast to events at metaphase I, sex differences in prophase I checkpoint control are less well studied. In males, problems in synapsis and/or recombination, arising either through chromosome abnormalities or targeted meiotic mutations, have variable effects on prophase I progression, ranging from normal germ cell development (Manterola et al. 2009) to complete pachytene loss (Burgoyne et al. 2009). In models where germ cell loss is observed, the effects are generally more severe in males than in females (Hunt and Hassold 2002; Kolas et al. 2004). This may be because in males, such defects disrupt Meiotic Sex Chromosome Inactivation (MSCI), the silencing of the X and Y chromosomes during prophase I (Mahadevaiah et al. 2008; McKee and Handel 1993). MSCI failure leads to misexpression of toxic sex-linked genes and subsequent midpachytene arrest (Royo et al. 2010). However, whether the prophase I checkpoint, like the SAC, is less robust in females than in males is unclear.

MSCI is a manifestation of a general mechanism, meiotic silencing, which acts in both sexes to inactivate genes on asynapsed chromosomes (Baarends et al. 2005; Turner et al. 2005). Although its purpose is unknown, meiotic silencing may serve a prophase I checkpoint function by starving germ cells of multiple essential gene products (Burgoyne et al. 2009). In view of its potential checkpoint function, there is a clear motivation for comparing meiotic silencing between males and females. Many components of the meiotic silencing pathway, including BRCA1 and γH2AFX, are observed on asynapsed chromosomes in both sexes (Baarends et al. 2005; Garcia-Cruz et al. 2009; Kouznetsova et al. 2009; Turner et al. 2005; Wojtasz et al. 2009). However, a recent study noted that the male meiotic silencing mark lysine-9 trimethylated histone H3 (H3K9me3) was absent on asynapsed chromosomes in the female (Taketo and Naumova 2013). Whether this and other epigenetic dissimilarities create sex differences in gene expression from asynapsed chromosomes is not known. Such an analysis requires single cell transcriptional approaches, in which expression at a given gene can be correlated with the synaptic status of the chromosome on which it resides. Gene-specific fluorescence in situ hybridization (RNA FISH) is especially useful for this purpose but has not yet been applied to mouse prophase I oocytes. We therefore used this technique to characterise meiotic silencing in the female and to compare it with that in the male.

## Results

### Asynapsed chromosomes exhibit sexually dimorphic epigenetic features

Prior to our transcriptional studies, we wished to examine epigenetic differences in asynapsed chromosome between males and females. The localization of many components involved in meiotic silencing in males has been examined in oocytes, including SYCP3, HORMAD1/2, BRCA1, ATR and γH2AFX (Baarends et al. 2005; Fukuda et al. 2010; Garcia-Cruz et al. 2009; Kouznetsova et al. 2009; Shin et al. 2010; Turner et al. 2005; Wojtasz et al. 2009). SYCP3 and HORMAD1/2 together act to recruit BRCA1 and ATR to asynapsed chromosome axes, after which ATR translocates through axis-associated loops, causing gene silencing through the creation of γH2AFX (Daniel et al. 2011; Kouznetsova et al. 2009; Royo et al. 2013; Turner et al. 2004; Wojtasz et al. 2012). We examined a further two silencing components, MDC1 and SUMO1, which also act as loop-associated silencing effectors (Ichijima et al. 2011; Rogers et al. 2004; Vigodner and Morris 2005), as well as H3K9me3, the latter of which has been found to be absent from asynapsed chromosomes in oocytes (Taketo and Naumova 2013). We used XO mice as our female model system, in order to directly compare the asynapsed X chromosome in oocytes with that in wild type (XY) spermatocytes. The asynapsed X chromosome in each sex was identified using an antibody to HORMAD2 (Wojtasz et al. 2009). At least 50 oocytes and spermatocytes were studied for each meiotic silencing factor assayed.

**Fig. 1:**
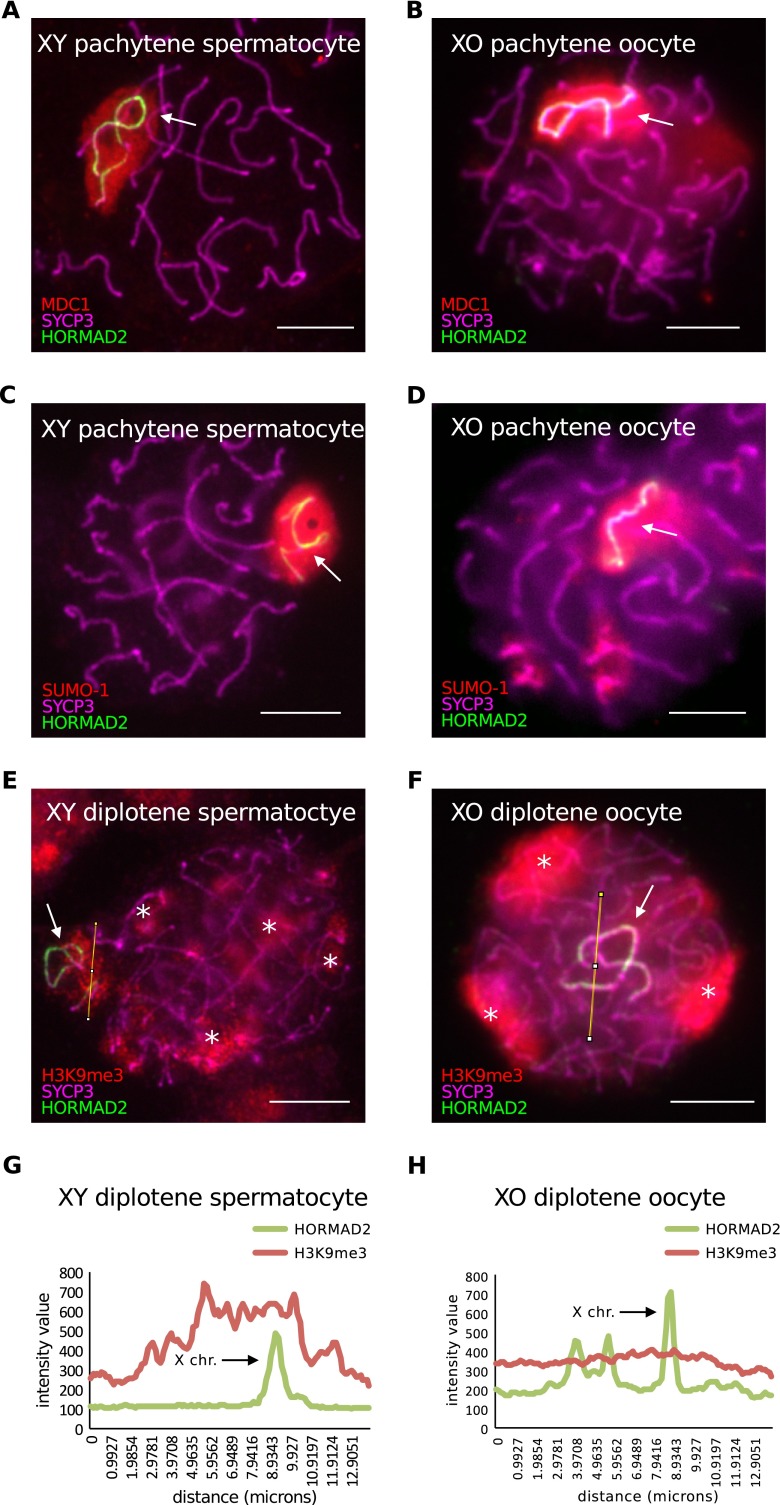
Analysis of epigenetic differences in meiotic silencing in oocytes compared to spermatocytes. **a** XY pachytene spermatocyte stained for SYCP3 (*magenta*), HORMAD2 (*green*) and MDC1 (*red*), showing MDC1 accumulation in the sex chromatin (*arrow*). **b** XO pachytene oocyte stained for SYCP3 (*magenta*), HORMAD2 (*green*) and MDC1 (*red*), also showing MDC1 enrichment on the asynapsed X chromosome (*arrow*). **c** XY pachytene spermatocyte stained for SYCP3 (*magenta*), HORMAD2 (*green*) and SUMO-1 (*red*), showing accumulation of SUMO-1 in the sex chromatin (*arrow*). **d** XO pachytene oocyte stained for SYCP3 (*magenta*), HORMAD2 (*green*) and SUMO-1 (*red*), also showing SUMO-1 enrichment on the asynapsed X chromosome (*arrow*). **e** XY diplotene spermatocyte stained with SYCP3 to mark chromosome axes (*magenta*), HORMAD2 (*green*) to label the asynapsed X and Y chromosomes (*arrow*) and H3K9me3 (*red*), which shows enrichment in the chromatin of the X and Y chromosomes and also at constitutive heterochromatin (*asterisks*). A line (*yellow*) was drawn through the asynapsed X (*arrow*) to quantify HORMAD2 and H3K9me3 intensities (**g**). **f** XO diplotene oocyte showing no enrichment of H3K9me3 in the chromatin of the asynapsed X chromosome (*arrow*). H3K9me3 staining is restricted to sites of constitutive heterochromatin (*asterisks*). A line (*yellow*) was drawn through the asynapsed X (*arrow*) to quantify HORMAD2 and H3K9me3 intensities (**h**). **g**, **h** Intensities of H3K9me3 and HORMAD2 immunostaining quantified by densitometry across the indicated paths along (**g**) the asynapsed X in the spermatocyte from panel **e**, and along (**h**) the asynapsed X in diplotene XO oocyte from panel **f**. *Scale bars* = 10 μm

We found that MDC1 and SUMO1 localised to the asynapsed X chromosome in XO females, as in XY males, during both pachynema and diplonema (Fig. 1a, b, 1a-d). In contrast, consistent with a previous study (Taketo and Naumova 2013), H3K9me3 patterns differed between the sexes (Fig. 1e, f). During pachynema, H3K9me3 was present both at centromeric heterochromatin and the asynapsed X chromosome in XY males. Enrichment of H3K9me3 at the asynapsed X chromosome was most clear during diplonema (Fig. 1e). However, during diplonema in XO females, H3K9me3 was enriched at centromeric heterochromatin but not on the asynapsed X chromosome (Fig. 1f). This sex difference in H3K9me3 was confirmed quantitatively (Fig. 1g, h). We conclude that the chromatin of asynapsed chromosomes exhibits sexually dimorphic epigenetic features.

### Meiotic silencing of genes on the asynapsed X chromosome is less efficient in females than in males

**Fig. 2:**
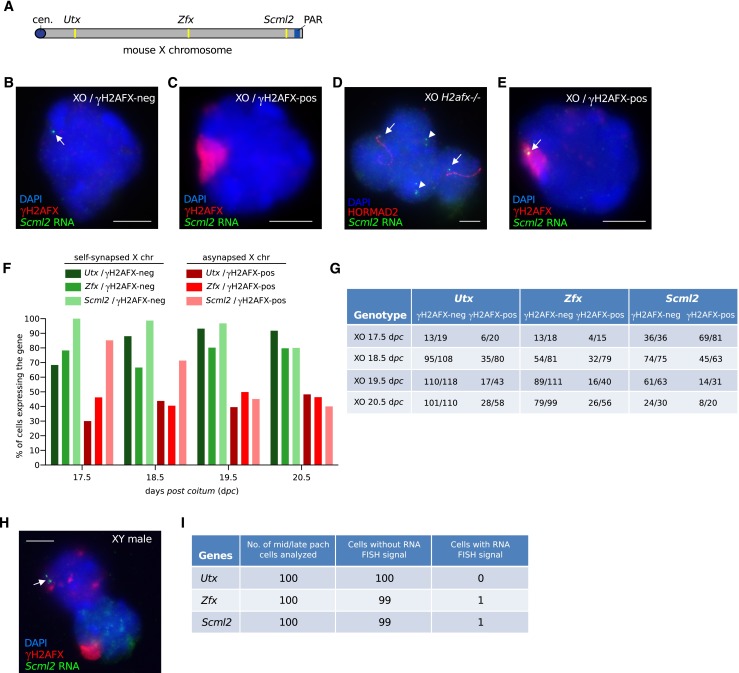
Incomplete silencing of the asynapsed X chromosome in XO oocytes. **a** Schematic of mouse X chromosome showing the location of three genes, *Utx*, *Zfx* and *Scml2*, which were used as RNA FISH probes to assess X chromosome transcription. PAR = pseudoautosomal region; cen. = centromere. **b** Control XO oocyte with a self-synapsed X chromosome (γH2AFX domain-negative) and an RNA FISH signal (*arrow*; *green*), indicating expression of the X-linked gene *Scml2*. Oocytes were distinguished from somatic cells based upon DAPI staining and nuclear morphology. **c** XO oocyte with an asynapsed X chromosome (γH2AFX domain-positive; *red*) and no RNA FISH signal, demonstrating silencing of *Scml2*. **d** Four adjacent XO *H2afx*−/− oocytes. The two middle nuclei have a self-synapsed X chromosome (HORMAD2-negative) and express *Scml2* (*arrowheads*). The two outside nuclei have an asynapsed X chromosome (HORMAD2-positive; *red*) and also express *Scml2* (*arrows*). Expression of *Scml2* was observed in all XO *H2afx*−/− oocytes with an asynapsed X chromosome (*n* = 34). **e** XO oocyte with an asynapsed X chromosome (γH2AFX domain-positive) with an RNA FISH signal (*arrow*), demonstrating expression of *Scml2*. **f** The percentage of XO oocytes expressing *Utx*, *Zfx* and *Smcl2* at 17.5, 18.5, 19.5 and 20.5 d*pc*. XO oocytes were subdivided into those without a γH2AFX domain, i.e. with a self-synapsed X chromosome (*green bars*), and those with a γH2AFX domain i.e. with an asynapsed X chromosome (*red bars*). One ovary was analysed for each gene and time point. **g** Raw data showing number of XO oocytes expressing *Utx*, *Zfx* and *Scml2* at 17.5, 18.5, 19.5 and 20.5 d*pc* out of the total number of oocytes analysed. **h** Robust silencing of X-genes in mid-late pachytene spermatocytes. Upper left nucleus: control zygotene spermatocyte with *Scml2* RNA FISH signal. Lower right nucleus: mid-late pachytene spermatocyte with γH2AFX-labelled sex body and no *Scml2* RNA FISH signal, indicating meiotic silencing. **i** Raw data showing counts of mid-late pachytene spermatocytes with and without RNA FISH signals for each probe. *Scale bars* = 5 μm

We next used gene-specific RNA FISH to assay meiotic silencing of the asynapsed X chromosome in XO oocytes. We performed RNA FISH for three X-linked genes, *Utx*, *Zfx* and *Scml2*. These genes are distant from each other on the X chromosome (Fig. 2a), and one, *Zfx*, has been shown to be essential for female fertility (Luoh et al. 1997). For each of the three genes, we carried out RNA FISH at four developmental time-points: 17.5, 18.5, 19.5 and 20.5 days *post coitum* (d*pc*). This allowed us to track X chromosome silencing all the way through pachynema and diplonema: at 17.5d*pc*, most oocytes are in pachynema, and by 19.5d*pc*, most oocytes are in late diplonema (Cloutier et al. 2015).

In approximately one half of oocytes from XO females, the single X chromosome self-synapses, and as a result, does not accumulate γH2AFX labelling (Turner et al. 2005). Cot-1 and RNA polII analyses have previously shown that in these oocytes, the X chromosome escapes meiotic silencing (Baarends et al. 2005; Turner et al. 2005). We therefore used XO oocytes with self-synapsed X chromosomes as internal, positive controls in our female RNA FISH experiments. Within this positive control oocyte population, all three X-genes were expressed at the expected high frequency, with the majority of oocytes showing an RNA FISH signal (Fig. 2b, green bars in Fig. 2f, quantitation in Fig. 2g). This high frequency of expression for each X-gene was observed at all four time-points analysed (green bars in Fig. 2f, quantitation in Fig. 2g).

Next, we assayed X-gene expression in XO oocytes with an asynapsed, γH2AFX-positive X chromosome. For all three genes, we found that the percentage of expressing oocytes was lower than that observed in XO oocytes with a self-synapsed X chromosome, thereby confirming the presence of meiotic silencing at all three loci (Fig. 2c, red bars in Fig. 2f, quantitation in Fig. 2g). In order to confirm that this meiotic silencing was dependent on H2AFX, we then performed RNA FISH for one of the X-genes, *Scml2*, on XO *H2afx* −/− females at 19.5d*pc* (Celeste et al. 2002). All XO *H2afx*−/− oocytes with an asynapsed X chromosome, identified by immunostaining for the asynapsis marker HORMAD2, expressed *Scml2* (34/34, i.e. 100 % oocytes expressing; Fig. 2d). This frequency of expression was higher than that observed in XO *H2afx* +/+ oocytes with an asynapsed X chromosome (only 14/31, i.e. 45 % oocytes expressing; Fig. 2c, f and g) and was similar to that in XO *H2afx* +/+ oocytes with a self-synapsed X chromosome (61/63, i.e. 97 % oocytes expressing; Fig. 2b, f and g). Thus, meiotic silencing in oocytes is *H2afx*-dependent.

Although meiotic silencing was clearly operating in XO females, we were surprised to find sizeable populations of oocytes with an asynapsed X chromosome in which *Utx*, *Zfx* and *Scml2* RNA FISH signals were present, despite the coexistence of γH2AFX silencing domains (Fig. 2e, red bars in Fig. 2f, quantitation in Fig. 2g). For example, at 19.5d*pc*, *Utx*, *Zfx* and *Scml2* were expressed in 39, 40 and 45 % of oocytes with an asynapsed X chromosome, respectively. This phenomenon was observed at all developmental time-points analysed (Fig. 2f, g). In order to ascertain whether this “leakiness” in meiotic silencing was specific to females, we then performed RNA FISH for *Utx*, *Zfx* and *Scml2* in wild type, XY males, focusing our analysis on mid-late pachytene spermatocytes. Silencing of each of the three X-genes in spermatocytes was highly efficient, with *Utx*, *Zfx* and *Scml2* RNA FISH signals present in 0, 1 and 1 % of spermatocytes, respectively (Fig. 2h, i). We conclude that meiotic silencing is less efficient in oocytes than in spermatocytes.

### Inefficient meiotic silencing in oocytes also affects asynapsed autosomes

XO females exhibit perinatal germ cell losses (Burgoyne and Baker 1985) that preferentially affect oocytes with asynapsed X chromosomes (Cloutier et al. 2015). We considered the possibility that silencing in some XO oocytes might be highly efficient, and that these oocytes were preferentially eliminated, and thus missing from our RNA FISH analysis. This was unlikely, because our XO experiments included oocytes harvested at 17.5 d*pc* (Fig. 2f, g) when germ cell elimination has not yet initiated in XO females (Burgoyne and Baker 1985). Nevertheless, to further exclude an effect of selection on our RNA FISH results, we performed RNA FISH on another model, in which the presence of an asynapsed chromosome does not elicit prophase I elimination. Tc1 females (O’Doherty et al. 2005) carry a copy of human chromosome 21 (h21), which self-synapses in 60 % of pachytene oocytes and is asynapsed in the remaining oocytes. Importantly, Tc1 oocytes with an asynapsed h21 chromosome persist from pachynema through diplonema (Cloutier et al. 2015).

**Fig. 3:**
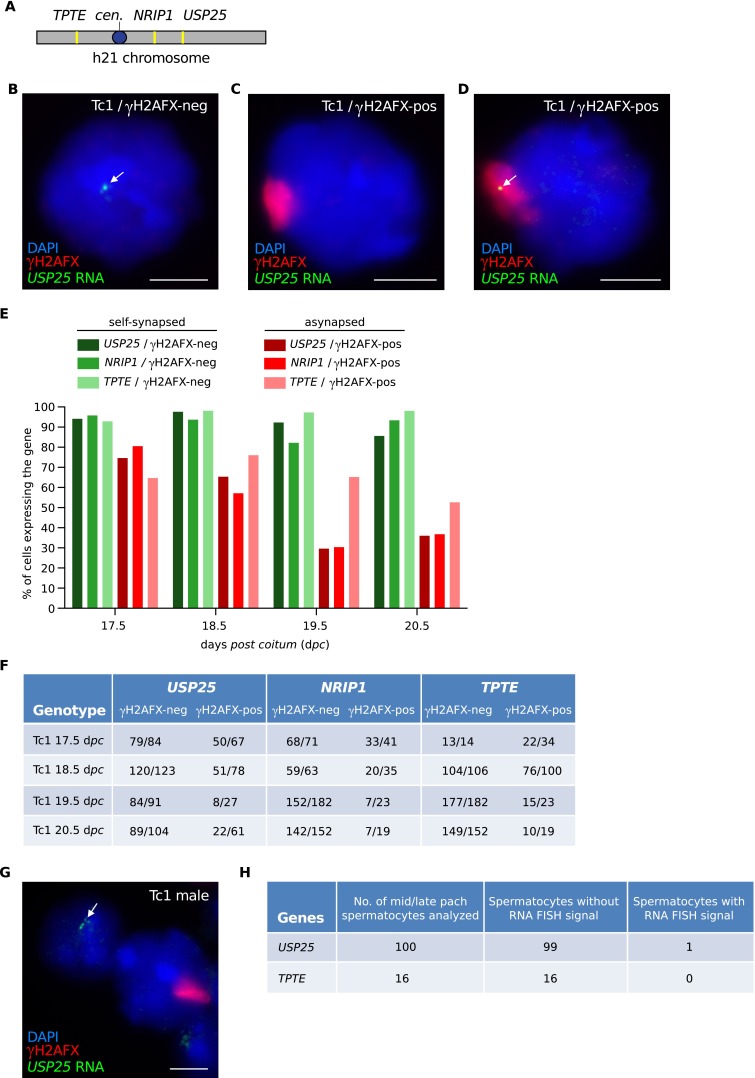
Incomplete silencing of the asynapsed h21 chromosome in Tc1 oocytes. **a** Schematic of Tc1 h21 chromosome showing the location of three genes, *TPTE*, *NRIP1* and *USP25*, which were used as RNA FISH probes to assess h21 chromosome transcription. cen. = centromere. **b** Control Tc1 oocyte with a self-synapsed h21 chromosome (γH2AFX domain-negative) and an RNA FISH signal (*arrow*; *green*), indicating expression of the X-linked gene *USP25*. **c** Tc1 oocyte with an asynapsed h21 chromosome (γH2AFX domain-positive; *red*) and no RNA FISH signal, demonstrating silencing of *USP25*. **d** Tc1 oocyte with an asynapsed h21 chromosome (γH2AFX domain-positive) and an RNA FISH signal (*arrow*), demonstrating expression of *USP25*. **e** The percentage of Tc1 oocytes expressing *USP25*, *NRIP1* and *TPTE* at 17.5, 18.5, 19.5 and 20.5 d*pc*. Tc1 oocytes were subdivided into those without a γH2AFX domain, i.e. with a self-synapsed Tc1 chromosome (*green bars*) and those with a γH2AFX domain, i.e. with an asynapsed X chromosome (*red bars*). One ovary was analysed for each gene and time point. **f** Raw data showing number of Tc1 oocytes expressing *USP25*, *NRIP1* and *TPTE* at 17.5, 18.5, 19.5 and 20.5 d*pc* out of the total number of oocytes analysed. **g** Robust silencing of Tc1 genes in mid-late pachytene Tc1 spermatocytes. Upper left nucleus: spermatid with *USP25* RNA FISH signal. Middle nucleus: mid-late pachytene spermatocyte with γH2AFX-labelled sex body and no *USP25* RNA FISH signal, indicating meiotic silencing. **h** Raw data showing counts of mid-late pachytene spermatocytes with and without RNA FISH signals for *USP25* and *TPTE. NRIP1* is not expressed in Tc1 testes. *Scale bar* = 5 μm

We performed RNA FISH on Tc1 oocytes for three genes, *USP25*, *NRIP1* and *TPTE*, which are located at different sites on the h21 chromosome (Fig. 3a). As with our XO experiments, we carried out RNA FISH for each of the three genes at four developmental time-points: 17.5, 18.5, 19.5 and 20.5 d*pc*. Tc1 oocytes with a self-synapsed h21 chromosome, which acted as RNA FISH positive controls, exhibited high frequencies of expression for each of the three genes, at all gestational ages (Fig. 3b, green bars in Fig. 3e, quantitation in Fig. 3f). Those with an asynapsed h21 chromosome exhibited lower expression frequencies, consistent with the presence of meiotic silencing (Fig. 3c, red bars in Fig. 3e, quantitation in Fig. 3f). However, reminiscent of our observations in XO females, in Tc1 females, many oocytes with asynapsed h21 chromosomes exhibited RNA FISH signals for the three genes studied. For example, at 19.5d*pc*, *USP25*, *NRIP1* and *TPTE* were expressed in 30, 30 and 65 % of oocytes with an asynapsed Tc1 chromosome, respectively (Fig. 3e, f). We then analysed the expression of the same genes in Tc1 males. *USP25* and *TPTE* were expressed in spermatogenic cells, while expression of *NRIP1* could not be detected. Importantly, in Tc1 pachytene cells with an asynapsed Tc1 chromosome, silencing of both *USP25* and *TPTE* was efficient (Fig. 3g, h). Thus, escape from silencing is not a feature specific to the XO female mouse model, nor indeed to the X chromosome.

### Meiotic silencing creates mosaic gene expression patterns in oocytes

**Fig. 4:**
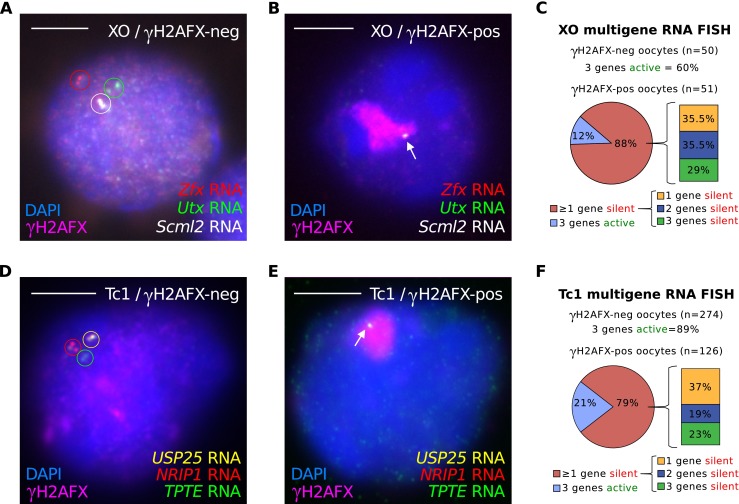
Mosaic silencing of asynapsed chromosomes in oocytes. **a**–**c** Simultaneous triple RNA FISH was performed in XO oocytes using probes for *Utx*, *Zfx* and *Scml2* at 19.5d*pc*. **a** Control XO oocyte with a self-synapsed X chromosome (γH2AFX domain-negative) and RNA FISH signals for all three genes (*circled*; *red*, *green* and *white*). **b** XO oocyte with an asynapsed X chromosome (γH2AFX domain-positive; *magenta*) and an RNA FISH signal only for *Scml2* (*arrow*), indicating that the silencing response is mosaic, inactivating two of the three genes analysed. **c** Quantitation of RNA FISH data. 60 % of γH2AFX-negative XO oocytes express all three genes simultaneously. The pie chart shows the percentage of XO oocytes with an asynapsed X chromosome that has at least one gene silenced (88 %). The accompanying bar chart shows the percentage of oocytes with one, two and three genes silenced. *n* represents the number of oocytes analysed from one 19.5 d*pc* ovary. **d**–**f** Simultaneous triple RNA FISH was performed in Tc1 oocytes using the probes for *USP25*, *NRIP1* and *TPTE* at 19.5d*pc*. **d** Control Tc1 oocyte with a self-synapsed h21 chromosome (γH2AFX domain-negative, *inset*) and RNA FISH signals for all three genes (*circled*), showing active transcription at all three loci. **e** Tc1 oocyte with an asynapsed h21 chromosome (γH2AFX domain-positive, *inset*) and only an RNA FISH signal for *USP25* (*arrow*), indicating that the silencing response is mosaic. **f** Quantitation of RNA FISH data. 89 % of γH2AFX-negative Tc1 oocytes express all three genes simultaneously. The pie chart shows the percentage of Tc1 oocytes with an asynapsed h21 that have at least one gene silenced (79 %). The accompanying bar chart shows the percentage of oocytes with one, two and three genes silenced. *n* represents the number of oocytes analysed from two 19.5 d*pc* ovaries. *Scale bars* = 5 μm. Note that RNA FISH signals appear in some cells appear as double dots and in others as single dots. At this stage of germ cell development, each locus will be comprised of two sister chromatids. *Double spots* most likely represent expression from sisters that are spatially separate, while *single spots* represent expression from sisters that are in close proximity to each other

We next questioned whether escape from meiotic silencing was concerted, affecting multiple genes on the same asynapsed chromosome simultaneously, or stochastic, affecting different genes independently of one another. To discriminate between these possibilities, we performed multicolour, triple RNA FISH for *Utx*, *Zfx* and *Scml2* in XO females at 19.5d*pc*. As expected, the majority of XO oocytes with a self-synapsed X chromosome exhibited RNA FISH signals for all three genes simultaneously (Fig. 4a; quantitation in Fig. 4c). Interestingly, however, in XO oocytes with an asynapsed X chromosome, different combinations of gene expression were observed. Twelve percent (*n* = 51) of oocytes with an asynapsed X chromosome expressed *Utx*, *Zfx* and *Scml2* simultaneously (Fig. 4c). However, within the remaining 88 % of oocytes, escape from silencing could be observed at none, at one, or at two of the X-genes studied, in roughly equal proportions. Thus, silencing across the X chromosome is stochastic. We observed the same phenomenon of stochastic gene silencing in Tc1 females using simultaneous triple RNA FISH for *USP25*, *NRIP1* and *TPTE* at 19.5 d*pc* (Fig. 4d–f). Thus, meiotic silencing creates mosaicism in gene expression patterns between oocytes.

### Gonadal sex determines male female differences in meiotic silencing

**Fig. 5:**
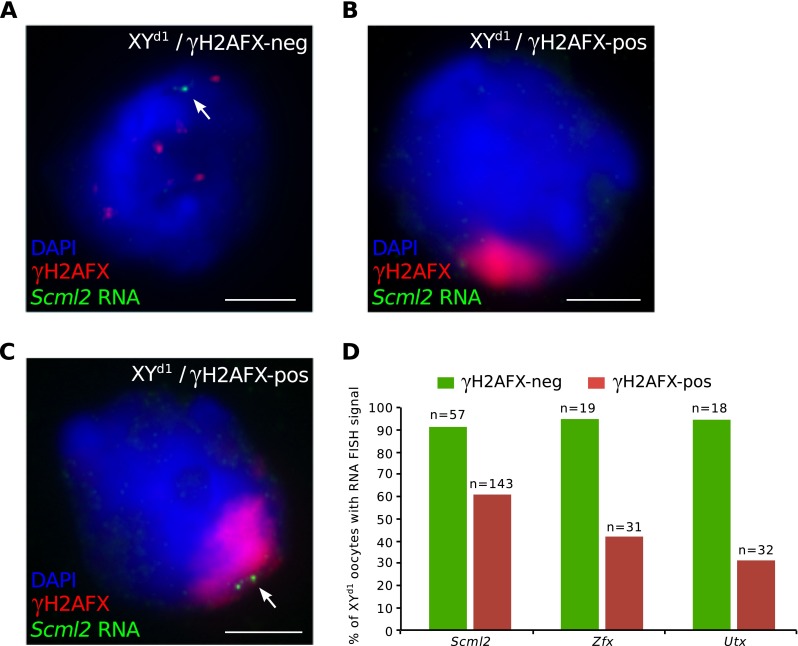
X chromosome silencing is inefficient in oocytes containing a Y chromosome. **a** Control XY^d1^ oocyte with no γH2AFX domain and an RNA FISH signal for *Scml2*. **b** XY^d1^ oocyte with a γH2AFX domain and no RNA FISH signal for *Scml2*, indicating silencing. **c** XY^d1^ oocyte with a γH2AFX domain and a RNA FISH signal for *Scml2*, indicating escape from silencing. **d** Graph of the percentage of XY^d1^ oocytes with an RNA FISH signal for *Scml2*, *Zfx* and *Utx*. XY^d1^ oocytes were subdivided into γH2AFX domain-negative (*green bars*) and γH2AFX-positive oocytes (*red bars*). *n* is the number of oocytes analysed from one 18.5 d*pc* ovary for each gene

Sexual dimorphisms can be controlled by male/female differences in sex chromosome genotype, e.g. the dose of X chromosomes, or the presence or absence of a Y chromosome. Alternatively, they can be regulated by the gonadal environment, i.e. the presence of a testis or an ovary (Arnold et al. 2012). We wished to establish whether sex chromosome complement or gonadal sex governed the sex difference in meiotic silencing efficiency that we had identified. Our data excluded an effect of X chromosome dose, because sex differences in meiotic silencing were observed in XO females and XY males, despite them both carrying a single X chromosome. However, a possible enhancing effect of the Y chromosome on meiotic silencing in males had not been considered. We therefore performed RNA FISH for the X-linked gene *Scml2* in sex-reversed XY^d1^ females (Mahadevaiah et al. 1998), in which oocytes contain an X and a Y chromosome. As in XO females, we observed escape of *Scml2* from silencing in XY^d1^ pachytene oocytes (81/143, i.e. 57 % oocytes expressing; Fig. 5a–d) at frequencies that far-exceeded those seen in XY males (Fig. 2h, i). Higher levels of escape from silencing in XY^d1^ oocytes compared to XY spermatocytes were also observed for *Utx* and *Zfx* (Fig. 5d). Thus, sex differences in meiotic silencing are driven by gonadal sex and not by sex chromosome constitution.

## Discussion

Chromosome abnormalities confer greater germ cell loss in males than in females (Burgoyne et al. 2009). This is due in part to the reduced stringency of the metaphase I spindle checkpoint in females (LeMaire-Adkins et al. 1997; Nagaoka et al. 2011) but is also thought to reflect ill-defined sex differences in the efficacy of the prophase I response to asynapsis (Hunt and Hassold 2002; Morelli and Cohen 2005; Nagaoka et al. 2012). Here we shed light on this sex difference by demonstrating that meiotic silencing in the female germ line is less efficient than in the male. Although the role of meiotic silencing in mammalian infertility is unclear, it may trigger prophase I elimination by rendering germ cells deficient in multiple gene products. Under this model, the detrimental effects of meiotic silencing would increase as a function of its efficiency, thereby providing an explanation as to why chromosome abnormalities cause more severe germ cell loss in males than in females.

Our multigene RNA FISH analysis shows that genes located on the same asynapsed chromosome are silenced to varying extents. In addition, the combination of genes that are silenced on a given asynapsed chromosome differs between oocytes. A potential caveat of this observation is that the efficiency of probe hybridization may be reduced when used in a multiplexing experiment. However, the mosaicism, assayed here at the level of nascent RNA, could create distinct gene expression profiles that disturb different biological pathways, both qualitatively and quantitatively. Thus, in XO females, and other chromosomally abnormal mouse models exhibiting prophase I germ cell losses, the precise cause of arrest could differ from oocyte to oocyte depending on the suite of genes that are silenced.

Why should meiotic silencing be more robust in males than in females? During male meiosis, the asynapsed X and Y chromosomes are transcriptionally inactivated by MSCI. Defects in MSCI cause complete midpachytene arrest, due to the misexpression of toxic sex-linked genes, e.g. *Zfy1* and *Zfy2* (Royo et al. 2010). We propose that meiotic silencing in males must be highly efficient in order to prevent the misexpression of these XY-encoded pachytene-lethal genes during normal male meiosis.

Although most components of the meiotic silencing pathway are conserved between the sexes, H3K9me3 is present on asynapsed chromosomes in the male but not in the female. Our data indicate that H2AFX phosphorylation creates mosaicism in gene expression patterns, while additional, male-specific chromatin changes, including H3K9me3, result in stable and complete silencing. Identification of the histone methyltransferases that catalyse H3K9 methylation on asynapsed chromosomes represents an important challenge in further understanding sex differences in the prophase I response to asynapsis. It will also be important to determine whether other factors involved in meiotic silencing in males (Becherel et al. 2013; Modzelewski et al. 2012) exhibit similar sexual dimorphisms.

## Materials and methods

### Animals

Females were set up in matings and checked daily for copulation plugs. The day of plugging was considered 0.5 days *post coitum* (d*pc*). Embryos were sacrificed at 17.5, 18.5, 19.5 and 20.5 d*pc* using UK Home Office Schedule I methods. Ovaries were dissected from embryos and flash frozen in liquid nitrogen. Material was stored at −80 °C until later use. XO mice were generated on a random bred MF1 background (NIMR stock) by mating XX females to fertile X^Y*^O males, which harbour an X chromosome fused with a Y chromosome and give rise to “O” gametes (Eicher et al. 1991). *H2afx−/−* mice (Celeste et al. 2002) were generated on the MF1 background. XO *H2afx−/−* mice were generated by crossing X^Y^O *H2afx+/−* males with XX *H2afx+/−* females. Tc1 mice (O’Doherty et al. 2005) were maintained on the MF1 background. XY ^d1^ females were produced on an MF1 background by mating XY males to sex-reversed XY^d1^ females (Mahadevaiah et al. 1998).

### Chromosome spreads and RNA FISH

Surface spreads were performed as previously described (Turner et al. 2004, 2005). Briefly, previously frozen (−80 °C) ovaries were macerated in Roswell Park Memorial Institute (RPMI) on Superfrost slides, cells were permeabilized for 10 min in 0.05 % Triton X-100 in distilled water and then fixed for 60 min in 2 % formaldehyde, 0.02 % SDS in phosphate-buffered saline (PBS). The slides were rinsed in distilled water, allowed to air dry and then blocked in PBT (0.15 % BSA, 0.10 % TWEEN-20 in PBS) for 60 min. Slides were incubated with the following antibodies in a humid chamber overnight at 37 °C: rabbit anti-SYCP3 (1:100, Abcam: ab15093), mouse anti-γH2AFX (1:100, Upstate: 16–193), guinea pig and rabbit anti-HORMAD2 (1:200, ref. Wojtasz et al. 2009), rabbit anti-H3K9me3 (1:100, Upstate 07–442), rabbit anti-SUMO1 (1:100, Abcam: ab32058) and sheep anti-MDC1 (1:10, Serotec: AHP799). Secondary antibodies (AlexaFluor 488, 594 and 647, Invitrogen) were applied 1:500 in PBS for 1 hour at 37 °C and mounted in Vectashield with 4′,6-diamidino-2-phenylindole (DAPI).

RNA FISH was carried out as previously described (Mahadevaiah et al. 2009). Briefly, previously frozen (−80 °C) ovaries were mascerated in RPMI on Superfrost slides, cells were permeabilized for 10 min in chilled CSK buffer (100 mM NaCl, 300 mM sucrose, 3 mM MgCl2, 10 mM PIPES, 0.5 % Triton X-100, 1 mM EGTA and 2 mM vanadyl ribonucleoside, pH 6.8) and then fixed for 10 min in chilled 4 % paraformaldehyde. Slides were then washed in PBS, dehydrated in a series of ethanol dilutions (2 × 70 %, 80 %, 95 %, 100 %) and air dried.

RNA FISH digoxigenin-labelled probes were prepared from 1 μg of BAC DNA (from CHORI: Scml2, RP24-204O18; Zfx, RP24-204018; USP25, RP11-296D11; NRIP1, RP11-22D1; from ABgene: TPTE, CTD-2260D15; Utx, gift from Mike Mitchell, University Marseilles) using the Biotin Nick Translation Kit (Roche), according to manufacturer’s instructions. For each probe, 100 ng digoxigenin-labelled BAC was prepared in 15 μl formamide (Sigma), with 3 μg mouse (for XO) or human (for Tc1) Cot1 DNA (Invitrogen) and 10 μg sheared salmon sperm DNA (Ambion). Probes were denatured for 10 min at 80 °C and combined with 15 μl pre-warmed (37 °C) 2× hybridization buffer (2× saline sodium citrate (SSC), 10 % dextran sulphate (Sigma), 1 mg/ml BSA and 2 mM vanadyl ribonucleoside) and incubated for 30 min at 37 °C. Finally, 30-μl pre-hybridized probes were applied to slides and incubated in a humid chamber overnight at 37 °C.

The next day, slides were washed at 42 °C, three times in 2× SSC and 50 % formamide, and three times in 2× SSC, for 5 min per wash. Slides were then transferred to 4× SSC and 0.1 % TWEEN-20, and then blocked (4× SSC, 4 mg/ml) bovine serum albumin and 0.1 % TWEEN-20) for 30 min in a humid chamber at 37 °C. Probes were detected using 30 μl of 1:10 anti-digoxigenin fluorescein, diluted in detection buffer (4× SSC, 1 mg ml-1 bovine serum albumin and 0.1 % TWEEN-20) for 60 min in a humid chamber at 37 °C.

Slides were washed three times for 2 min in 4× SSC and 0.1 % TWEEN-20. For subsequent immunofluorescence, 50 μl of primary antibody against γH2AFX (Upstate, 16–193), diluted 1:100 in 4× SSC and 0.1 % TWEEN-20, was added to slides and incubated for 30 min in a humid chamber at room temperature. Slides were washed for 2 min in 4× SSC and 0.1 % TWEEN-20. Next, 50 μl of secondary antibody (AlexaFluor 594 conjugated), diluted 1:100 in 4× SSC and 0.1 % TWEEN-20, was added to slides and incubated for 30 min in a humid chamber at room temperature. Finally, slides were washed for 2 min in 4× SSC and 0.1 % TWEEN-20 and mounted in Vectashield with DAPI.

For RNA FISH analyses, cells were first categorised based upon the presence or absence of a γH2AFX domain or HORMAD2. The cells were then classified based upon the presence or absence of RNA FISH signals.

### Imaging

Imaging was performed using an Olympus IX70 inverted microscope with a 100-W mercury arc lamp. For chromosome spread and RNA FISH imaging, an Olympus UPlanApo 100×/1.35 NA oil immersion objective was used. For ovary section imaging, an Olympus UPlanApo 20×/0.75 NA objective was used. A Deltavision RT computer-assisted Photometrics CoolsnapHQ CCD camera with an ICX285 Progressive scan CCD image sensor was utilised for image capture. 16-bit (1024 × 1024 pixels) raw images of each channel were captured and later processed using Fiji.
